# Tumor resection in glioblastoma mouse models: Surgical techniques and translational potential

**DOI:** 10.1093/noajnl/vdag044

**Published:** 2026-02-16

**Authors:** Julie Van Nieuwenhuyze, Aaron Ziani Zeryouh, Stéphanie De Vleeschauwer, Matteo Riva, An Coosemans

**Affiliations:** Laboratory of Tumor Immunology and Immunotherapy, Department of Oncology, Leuven Cancer Institute, KU Leuven, Belgium; Laboratory of Tumor Immunology and Immunotherapy, Department of Oncology, Leuven Cancer Institute, KU Leuven, Belgium; Laboratory Animal Center KU Leuven, Leuven, Belgium; Laboratory of Tumor Immunology and Immunotherapy, Department of Oncology, Leuven Cancer Institute, KU Leuven, Belgium; Department of Neurosurgery, Centre Hospitalier Universitaire (CHU) UC Louvain Namur, University Hospital of Godinne, Yvoir, Belgium; Laboratory of Tumor Immunology and Immunotherapy, Department of Oncology, Leuven Cancer Institute, KU Leuven, Belgium; Department of Neurosurgery, Centre Hospitalier Universitaire (CHU) UC Louvain Namur, University Hospital of Godinne, Yvoir, Belgium

## Abstract

Surgical resection is a cornerstone of glioblastoma (GBM) treatment in patients; yet, it remains underrepresented in preclinical studies with mouse models. This narrative review analyzes the current resection techniques used in mouse GBM studies and evaluates their feasibility, reproducibility and translational relevance. A total of 27 eligible studies were included in the review and categorized by resection method: non-guided biopsy punch, non-guided freehand resection, fluorescence-guided freehand resection (using fluorescent cell lines or dyes), and magnetic resonance imaging (MRI)-guided approaches. While non-guided techniques (biopsy punch or freehand) are commonly employed due to their simplicity and affordability, they lack the precision and adaptability of more advanced methods. Fluorescence-guided resection improves tumor visualization but requires costly equipment and can introduce immunological biases. So-called MRI-guided strategies remain rare, and MRI is not actually used intraoperatively. Despite technically challenging, GBM surgical resection in mice is feasible and several effective techniques are currently available. The systematic implementation of tumor resection in GBM preclinical studies could allow to more closely mimic the human scenario thereby improving the predictive value of GBM animal experiments.

Key Points

- Surgical resection in mouse GBM models is feasible but underused.
- Non-guided methods are simple, yet lack precision and reproducibility.
- Fluorescence-guided resection improves tumor visibility in mouse GBM models.

Glioblastoma (GBM) is the most common malignant primary brain cancer.^1^^,^^2^ The standard treatment includes maximal surgical resection, followed by radiotherapy and chemotherapy with the alkylating agent Temozolomide (TMZ). Despite this multimodal approach, recurrence is almost inevitable, resulting in a poor prognosis with a median survival of approximately 15 months.^3–5^

Surgical resection remains the cornerstone of the standard of care (SoC) for GBM. Several intraoperative imaging techniques are used to improve tumor visualization and increase the extent of resection, ultimately influencing survival.^6^ 5-Aminolevulinic acid (5-ALA)-based fluorescence-guided surgical resection is performed in most of cases, since it has shown to improve survival compared to standard non-fluorescence guided surgery.^7^ Other strategies include intraoperative MRI, CT scan, and ultrasonography.^8^

In addition to its well-documented effects in reducing tumor burden, surgical resection of GBM has been shown to modulate the immune landscape of the tumor microenvironment. However, studies report conflicting outcomes regarding whether this modulation leads to immune stimulation or suppression. Some findings suggest that resection may enhance antitumor immune responses by alleviating local immunosuppression, while others indicate it may dampen immunity due to surgical stress, disruption of immune cell infiltration, or the release of immunosuppressive factors.^9^^,^^10^

Despite the clear importance of tumor resection for GBM patients, also in view of immunotherapy combination, this therapy is rarely reflected in preclinical research. Neverthe­less, developing effective GBM resection models and integrating such treatment in the experimental design represents a fundamental step to reduce the well-known translational gap between preclinical GBM studies and GBM patients. By critically examining the available resection techniques for preclinical GBM models and their translational relevance, this review aims to stress the need for a more clinically relevant preclinical approach that better reflects current SoC, thereby improving the predictive value of preclinical research in this area.^11^

## Purpose of the Review

In this narrative review, we will identify and compare different resection techniques currently available for GBM mouse models focusing on their feasibility, cost-effectiveness, and overall impact on research outcomes.

## Methods

A PubMed search was conducted to identify studies on preclinical GBM resection methods. The search strategy used the following terms: (“high grade glioma” OR “glioblastoma” OR “GBM” OR “brain tumor”) AND (“surgical resection”) AND (“mouse model” OR “preclinical models”). These terms were combined using Boolean operators to ensure a comprehensive search.

The initial selection was based on title and abstract screening. Relevant publications were compiled into a database (Excel 2013, Microsoft, Redmond, Washington), and full texts were subsequently analyzed.

Inclusion criteria were studies published in English and those utilizing tumor resection in mouse models. Exclusion criteria included non-English studies, studies involving animal species other than mice, and review or meta-analysis articles.

## Results

### Literature Search

**Figure 1.**
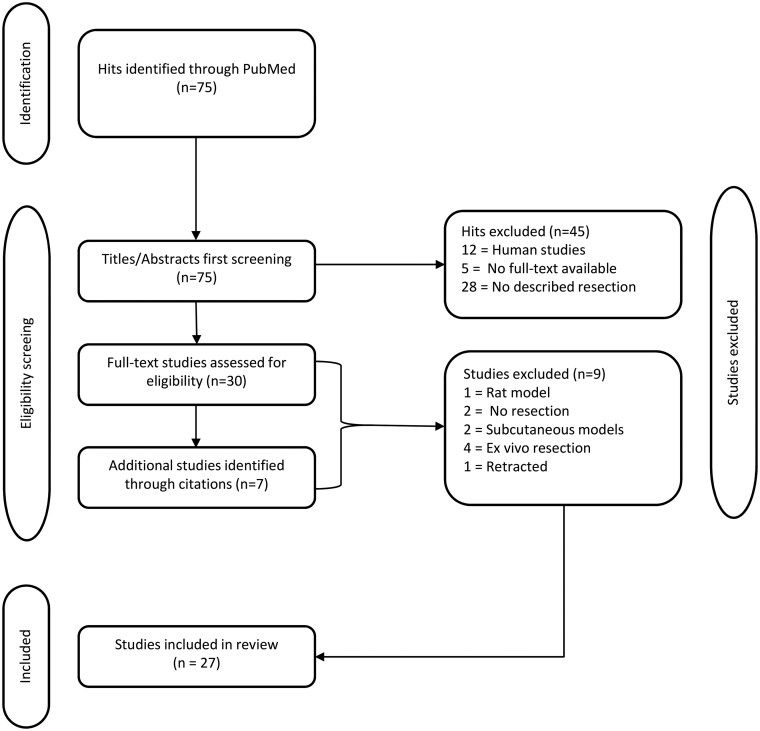
Study selection process for preclinical glioblastoma resection models. Note. Flowchart detailing study selection for the review of preclinical glioblastoma resection models.

The PubMed search identified 75 studies. In total, 45 studies were excluded after analyzing abstracts since the surgical resection of the brain tumors was actually not employed or they involved other species than mice. Consequently, 30 studies were full-text analyzed. The additional search within the reference list of those 30 articles yielded seven more relevant studies. In total, 37 articles underwent full-text review. Of these, nine studies were then excluded due to the absence of orthotopic models, the reliance on *ex vivo* resection methods, the use of non-mouse-based models, or the absence of surgical resection as experimental method. One study was excluded because it had been retracted during the preparation of the review. At the end of the selection process (Figure 1), 27 studies were considered eligible for this review.

### Resection Methods for GBM Mouse Models

Included preclinical studies were classified based on their approach for tumor tissue identification (non-guided, fluorescence-guided, and image-guided) and technique of resection (biopsy-punch, free-hand resection). Study classification is summarized in Table 1.

**Table 1.** Techniques screened for preclinical glioblastoma resection

Surgical technique	Author	Year	Strain	Sex	Age (in weeks)	Cell line	Description	Efficacy of resection	Post-operative outcomes	Recurrence
Biopsy punch	Bianco et al^12^	2017	Specific opportunistic pathogen-free NMRI nude mice	Female	6	U87 MG	Punch biopsy resection was performed by inserting a 2 mm biopsy punch 3 mm deep into the exposed tumor tissue, followed by twisting to excise the sample before removal with a Pasteur pipette connected to a diaphragm vacuum pump.	Subtotal resection	Tumor resection was well-tolerated, and led to a significantly prolonged OS.	Recurrence was observed in all resected animals.
	Zhao et al^13^	2018	NRMI nude mice	Female	6	U87 MG	Punch biopsy resection was performed by inserting a 2 mm biopsy punch 3 mm deep into the exposed tumor tissue, followed by twisting to excise the sample before removal with a Pasteur pipette connected to a diaphragm vacuum pump.	Subtotal resection	Median survival was significantly prolonged in resected mice	Recurrence was observed in all resected animals.
	Bastiancich et al^14^	2017	NRMI nude mice	Female	6	U87 MG	Punch biopsy resection was performed by inserting a 2 mm biopsy punch 3 mm deep into the exposed tumor tissue, followed by twisting to excise the sample before removal with a Pasteur pipette connected to a diaphragm vacuum pump.	Subtotal resection	Median survival of resected mice was prolonged with 11.5 days compared to control mice.	Recurrence was observed in all resected animals.
	Gazaille et al^15^	2023	NRMI nude mice	Female	6	U87 MG	Punch biopsy resection was performed by inserting a 2 mm biopsy punch 3 mm deep into the exposed tumor tissue, followed by twisting to excise the sample before removal with a Pasteur pipette connected to a diaphragm vacuum pump.	Subtotal resection	Median survival of resected mice was 38 days.	Recurrence was observed in all resected animals.
	Schiapparelli et al^16^	2020	Athymic nude mice	Male	6	GBM1A-Luc-GFP	A 3.0-mm biopsy punch was used to mark the resection area on the brain surface, after which tumor tissue was carefully debulked with microscissors to a depth of ∼1.8 µm along the defined edges.	97.4 % total gross resection rate was achieved.	/	Recurrence was observed in all resected animals after 1 week.
Non-guided free-hand resection	Chen et al^17^	2022	1. C57BL/6 mice 2. huHSC-NOG-EXL mice	/	6-8	1. GL261-Luc 2. Patient-derived glioma cells	Surgical resection was performed under microscopy at x20 magnification.	Resection removed bulk tumor, residual cells remained.	The resection cavity became an immunosuppressive niche due to the influx of immunosuppressive macrophages and microglia.	Recurrence occurred in all resected animals.
	Liu et al^18^	2021	Kumming mice	Male	Adult	G422TN	The tumor mass was macroscopically completely removed with micro-forceps under a stereomicroscope.	Subtotal resection	Less than 5% surgical death occurred within 48 h after the surgery. Resection prolonged overall survival.	Tumor recurred macroscopically within 3-5 days
	Kang et al^19^	2023	C57BL/6 mice	Male	8	U87 MG-Luc	The resection surgery consisted of tumor removal with a surgical drill, followed by suction of the resected tumor.	Resection removed bulk tumor, residual cells remained.	Median survival of resected animals was 19.5 days	Tumor recurrence occurred starting form 1 week after resection.
	Otvos et al^20^	2021	C57BL/6J mice	Male	6	GL261 and NS/CT-2A	The visible tumor mass was debulked using suction, retraction, and microdissection until only normal brain tissue was observed.	Resection removed bulk tumor, residual cells remained.	Tumor resection prolonged median survival with 4 days compared to control mice Resection led to a decrease in peripheral T cells and an increase in gMDSC.	Recurrence was observed in all resected animals
	Sun et al^21^	2023	C57BL/6J mice	Female	6-8	GL261-Luc	Tumor tissue was excised using a microcurette and scalpel under microscopic guidance.	Subtotal resection	/	Tumor size was markedly increased 2 weeks after resection.
Cell-line based fluorescence-guided resection	Sheets et al^22^	2020	Athymic nude mice	/	Adult	PDX-Fluc-GFP	Tumor resection was performed by gentle aspiration with a vacuum pump under fluorescence stereomicroscope guidance until no fluorescent signal remained.	Subtotal resection	Median survival of resected mice was 31 days post-resection.	Tumor recurrence occurred 3 weeks post-resection.
	Graham-Gurysh et al^23^	2018	Nude BALB/c mice	/	/	U87 MG-FLuc-mCherry	Tumor resection was performed by gently aspiration under fluorescence guidance.	The average percentage of tumor resected for all mice was 95.4 ± 9%	Median survival of resected mice was estimated 22 days.	All resected animals relapse within 10 days post-resection, tumor regrew at exponential rates.
	Okolie et al^24^	2016	C57BL/6 mice	/	/	TRP-mCherry-FLuc allographts	Tumors were surgically excised under fluorescence microscopy using a combination of aspiration and microsurgical dissection.	Resection achieved > 90% reduction n tumor volume	Resection significantly prolonged overall survival. Resection accelerated tumor growth and invasion, driven by injury-induced reactive astrocytes that promoted proliferation and migration.	Recurrent tumor was visible near the resection cavity 5 days after resection.
	Choi et al^10^	2017	C57BL/6 mice	Female	8-9	NS/CT-2A-FLuc-mCherry and GL261-FLuc-mCherry	Fluorescence-guided mechanical resection was performed under stereomicroscope using a Leica surgical microscope at ×20 magnification, debulking tumors to the tissue interface.	Subtotal resesction	Resection significantly prolonged overall survival. Tumor resection led to an increase in M1/M2 ratio + resection reduced MDSC and enhanced infiltration of dendritic cells and T cells into the tumor in the NS/CT-2A model.	Recurrence occurred in all resected animals
Cell-line based fluorescence-guided resection	Katta et al^25^	2019	Athymic nude mice	/	/	U251-Luc-RFP	Brain tumors and the surrounding vasculature were first imaged using a commercial fluorescent microscope and then by a custom benchtop OCT imaging system. After tumor pre-imaging, brain tumors were laser-ablated under OCT guidance.	Subtotal resection	Residual damage after cutting appears as a ring of higher density around the ablation crater, corresponding to a rim of thickened tissue.	/
	Fan et al^26^	2018	NOD SCID-IL2R-γ chain-deficient mice	/	6-8	U87 MG-Luc-GFP	Tumor resection was performed using a laser fiber probe guided by semi-quantitative BLI, starting at the boundary with low power and short duration. Power and probe parameters were gradually increased towards the center until tumorous tissue disappeared.	The resection ratio reached over 99%.	Four out of six mice died in the early postoperative period. The mortality rate of non-resected mice was significantly higher than that of resected mice.	Recurrence was observed in all resected animals.
	Kauer et al^27^	2011	Athymic nude mice	/	6-8	U87-FLuc-mCherry	Fluorescence-guided mechanical resection was performed under stereomicroscope using a Leica surgical microscope at ×20 magnification, debulking tumors to the tissue interface.	More than 60% of the tumor was resected in mice bearing small tumors, whereas more than 80% of the tumor was resected in mice with large tumors.	Resection significantly prolonged overall survival.	Recurrence was observed in all resected animals.
	Bagό et al^28^	2016	Athymic nude mice	/	6-8	U87-FLuc-mCherry	Tumor resection was performed under mCherry fluorescence using a stereotactic frame, Olympus MVX-10 microscope, and Hamamatsu ORCA-03G CCD camera, with surgical dissection and aspiration of the tumor tissue.	Subtotal resection	/	/
Cell-line based fluorescence-guided resection	Redjal et al^29^	2015	Athymic nude mice	/	4-6	Fluc-mCherry patient-derived GBM cells	Tumor resection was performed under mCherry fluorescence using a stereomicroscope.	Subtotal resection	/	/
	Sheets et al^30^	2018	Athymic nude mice	/	/	U87-FLuc-mCherry	Tumor visualization was performed with a stereomicroscope, and resection achieved by aspirating fluorescent tissue using a vacuum pump with a pipette tip until no signal remained.	Resection removed majority of tumor, residual cells remained.	/	/
	Hingtgen et al^31^	2013	Athymic nude mice	/	6-8	U87-Fluc-GFP	Tumor visualization and resection were performed under an Olympus SZX10 stereomicroscope with DP-72 camera and CellSens software, using GFP fluorescence to identify the xenograft. The U87-GFP-FLuc tumor was excised by surgical dissection and aspiration.	Greater than 90% removal of the tumor, only small residual tumor deposits remaining at the border of the resection cavity	Resection significantly prolonged overall survival.	Recurrence was observed from day 5 post-resection. The recurrent tumor was anatomically distinct from the primary tumor: recurrent tumors exhibited a faster growth rate and 46% reduction in blood vessel density.
Standard fluorescence-guided resection	Ziani-Zeryouh et al^32^	2024	C57BL/6 mice	Female	12-14	NS/CT-2A	Tumor visualized using intravenous fluorescein under UV light with a GFP filter. All visible tumor tissue was carefully removed using a suction pump with a sterile 200 μL tip.	/	Resection caused mortality in 20% of mice.	/
	Le Reste et al^33^	2020	C57BL/6rJ mice	Male	8	GL261-Luc	Tumor visualized using intravenous fluorescein and UV light. The resection was carried out using a surgical microscope, and the tumor was gently aspirated with a small suction device.	Subtotal resection	Resection did not have a significant survival impact.	Recurrence occurred in all resected animals. Recurrent tumors showed higher infiltrative capacity.
Near-infrared fluorescence-guided resection	Kurbegovic et al^34^	2021	Rj: NMRI-Foxn1^nu/nu-^ mice	Female	7-10	U87 MG-Luc2 and PDX	Fluorescence-guided surgery was performed after administration of NIR-probe IRDye800CW-AE344, targeting uPAR in GBM cells, and 5-ALA. All animals underwent 5-ALA–guided resection, while two received additional NIR-guided surgery. With NIR assistance, residual tumor was identified and removed until minimal or no signal remained.	Complete resection with the NIR-probe is claimed	The NIR-probe is superior for tumor resection to 5-ALA, as less residual tumor was shown with the probe.	/
	Li et al^35^	2018	athymic BALB/c nude mice	Male	4-5	U87 MG-Fluc-GFP	De NIR probe MMP-750, targeting MMP in GBM, was administered to perform both fluorescence molecular imaging (FMI) and fluorescence molecular tomography (FMT) in mice. Real-time intraoperative fluorescence image-guided surgery was performed under a fluorescence stereomicroscope.	Complete resection is claimed: no GFP signal nor BLI signal left	/	/
Near-infrared fluorescence-guided resection	Li et al^36^	2018	athymic BALB/c nude mice	Female	5	U87 MG	The 68Ga-IRDye800CW-BBN PET/NIR dual-modality imaging probe, targeting GRPR in GBM, was administered intravenously to perform image-guided resection.	Complete resection with the NIR-probe is claimed	/	/
Magnetic resonance imaging-guided resection	Oudin et al^37^	2024	Athymic nude mice	Female	/	PDX	Surgical resection in the mouse brains was performed based on MRI-guided coordinates. Bonn Micro probes were used to delineate the border and the depth of the resection site. A needle was gently moved in the brain to destroy the tumor-containing tissue before aspiration with a small glass Pasteur pipette.	Near total (80-90% tumor core removed) to complete total (approximately 100% tumor core removed) extent of resection was achieved.	Resection significantly prolonged overall survival.	Recurrence was observed in all resected animals. The recurrent tumor developed within the same cavity and displayed histopathological features comparable to the primary tumor.

*Note.* Overview of techniques screened for preclinical glioblastoma resection, including non-image-guided methods, fluorescence-guided methods, and MRI-guided methods. (5-ALA: 5 aminolevulinic acid, BLI: Bioluminescent imaging, FLuc: Firefly luciferase, GBM: Glioblastoma, GFP: green-fluorescent protein, gMDSC: granulocytic MDSC, GRPR: gastrin-releasing peptide receptor, Luc: luciferase, MDSC: Myeloid-derived suppressor cell, MMP: metalloprotease, MRI: Magnetic resonance imaging, NIR: Near-infrared, OS: Overall survival, PDX: Patient-derived xenograft, RFP: Red-fluorescent protein, uPAR: urokinase-type plasminogen activator receptor).

#### Non-guided techniques

##### Biopsy Punch

First introduced by John Bianco et al in 2017,^12^ the method has gained popularity due to its relative ease, speed, and suitability for evaluating localized therapeutic interventions. Five studies using this technique were included in this review, four of which originated from the same research group, meaning only two independent groups have reported its use.^12–16^

**Figure 2.**
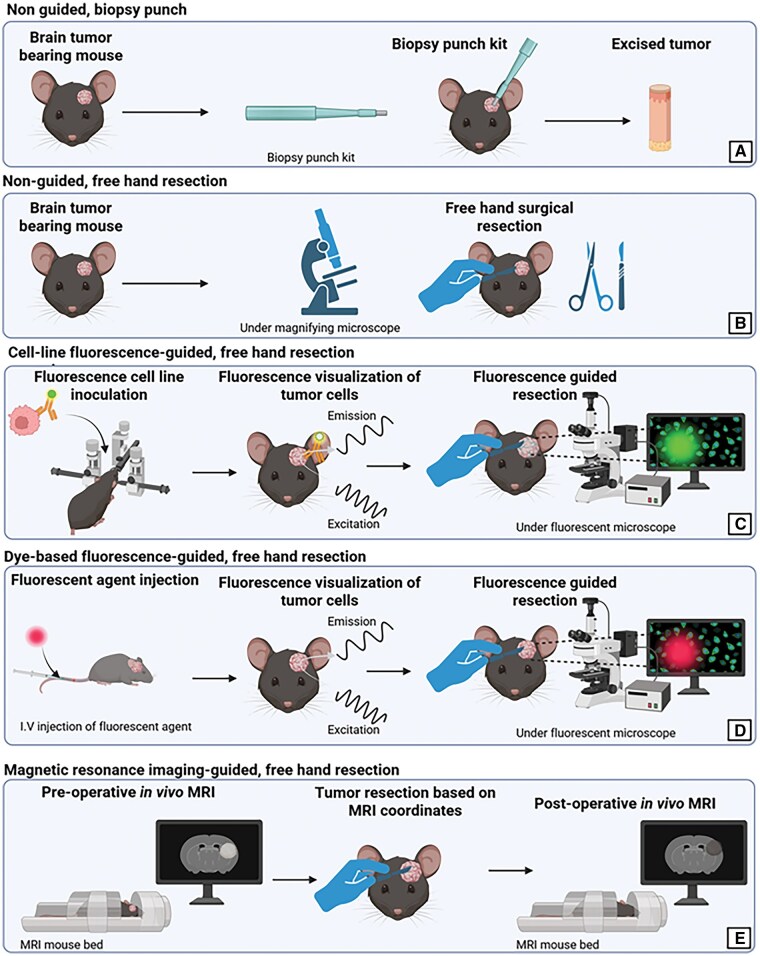
Resection techniques for preclinical glioblastoma models. *Note*. Overview of resection techniques for preclinical glioblastoma models. (A) Biopsy punch technique: Tumor removal using a biopsy punch kit. (B) Free hand resection: Surgical removal of tumors using standard surgical instruments. (C) Cell-line fluorescence-guided resection: Tumor resection guided by fluorescence-labeled cell lines for enhanced tumor visualization; resection under microscope. (D) Dye-based fluorescence guided tumor resection: Fluorescent agents enable real-time visualization of tumor for precise resection under a microscope. (E) Magnetic resonance imaging-guided resection: pre- and post-operative MRI imaging provides spatial guidance for accurate tumor removal.

The biopsy punch technique is a streamlined method for tumor resection in preclinical GBM models, relying on a standardized cylindrical tool (7 mm long, 2 mm Ø, KAI Medical, Germany) to excise tumor tissue with precision (Figure 2).^12–15^ After creating a cranial window, the punch is inserted to a depth of 3 mm and rotated to remove the tumor mass through vacuum aspiration.^12–15^ No post-resection analgesics were administered.^12^ In contrast, Schiapparelli et al employed a 3.0-mm biopsy punch to delineate the resection area.^16^ Using microscissors, the tumor was debulked within the boundaries defined by the biopsy punch, to a depth of approximately 1.8 µm.^16^ After resection, animals received a subcutaneous injection of buprenorphine.^16^

This approach does not require the use of a magnifying microscope, making the setup and execution even more straightforward. Typically taking only 5-10 min per surgery, the technique allows for consistent removal of tissue across animals, reducing operator variability and improving reproducibility between experiments. The punch technique produces a clean, well-defined cavity. All studies reported subtotal tumor resections, yet near-complete tumor removal was achieved, as evidenced by a 97.4% gross total resection rate reported by Schiapparelli et al.^12–16^

Bianco et al reported that the tumor resection was well-tolerated and did not cause any deleterious neurological deficits.^12^ However, swelling of the brain parenchyma and excessive bleeding, of which the extent varied depending on tumor location and vascular anatomy, was observed in all animals undergoing the procedure, though it typically subsided within 2-3 min.^12^ Bianco et al state that mortality and morbidity were not observed during the biopsy punch resection procedure.^12^ Surgery-related morbidity and mortality are not mentioned in the other publications.

Resection performed by punch biopsy was able to prolong survival, however, recurrence was observed in all resected mice.^12–16^ According to Schiapparelli et al, tumor recurrence was detected by bioluminescent imaging (BLI) starting from 1 week after resection, with complete refilling of the resection cavity by 1 month.^16^ In addition, Bianco et al found that recurrence was robust and aggressive, and frequently extended beyond the resection cavity into the surrounding brain tissue.^12^

##### Free-Hand Resection

Five studies using this technique were included in this review.^17–21^ Freehand tumor resection in preclinical GBM models involves the manual excision of the tumor mass under direct visual guidance using a magnifying microscope or loupes (Figure 2). A cranial window is created around the original injection site to fully expose the tumor. The surgeon then proceeds to excise the tumor using micro-forceps, suction devices, or a combination of both, continuing until the surgical cavity reveals white, healthy brain parenchyma, which serves as a visual indicator of the appropriate resection boundaries. The procedure typically takes between 15 and 30 min, depending on the size and accessibility of the tumor. Although techniques may vary slightly across studies, no major differences in outcome have been reported based on tool selection alone. However, reports of pre-operative morbidity and mortality are lacking for most studies except for Liu et al reporting a < 5% surgical death within 48 h after surgery. Only two studies reported details on post-operative care. In the study by Kang et al, mice received 5% glucose supplementation following resection.^19^ In the study by Otvos et al, post-operative care included daily subcutaneous saline injections for hydration over 4 days.^20^ Analgesia was provided with buprenorphine administered immediately after surgery, again 4-6 h post-operatively, and on the following morning.^20^ In addition, neomycin and ibuprofen were supplied via the drinking water.^20^ Four studies reported the occurrence of bleeding during resection.^18–21^ Liu et al used gelatin foam to control bleeding, while Kang et al reported that hemostasis was achieved but did not provide further details on the method used.^18^^,^^19^ In the study by Otvos et al, bleeding was managed through copious irrigation in combination with an oxidized regenerated cellulose matrix.^20^ Sun et al achieved hemostasis by applying sterile gauze.^21^ Free-hand tumor resection aims to replicate the clinical concept of maximal safe resection by removing as much tumor as possible while preserving surrounding healthy tissue. One key advantage is its flexibility as it can be adapted to tumors of varying sizes, shape and locations, and it allows the surgeon to attempt a more extensive and tailored resection, closer to real-world surgical conditions.

A mild improvement of survival following resection was reported by Liu et al, Kang et al and Otvos et al.^18–20^ In all studies, only subtotal resection was achieved, with residual cells remaining, and recurrence was consistently observed using either BLI or MRI. Liu et al observed macroscopic tumor recurrence within 3 days after resection.^18^ Tumor recurrence after resection was positively correlated with Akt activation, which in turn upregulated programmed death-ligand 1 (PD-L1) and vimentin, promoting proliferation and migration of recurrent tumor cells and potentially contributing to early recurrence.^18^ Kang et al reported tumor recurrence from 1 week after resection, with a large tumor visible within 2-3 weeks on BLI.^19^ Finally, Sun et al reported a marked increase in tumor size approximately 2 weeks after resection on BLI.^21^

Three studies also report some immunological effects of the resection. Chen et al demonstrated that the resection cavity became an immunosuppressive niche due to the influx of immunosuppressive macrophages and microglia.^17^ In addition, Liu et al observed PD-L1–expressing astrocytes surrounding tumor-infiltrating foci at the invasive front, indicating that astrocytes might contribute to immune suppression during GBM recurrence following surgery.^18^ Moreover, Otvos et al demonstrated that surgical resection induced systemic immune suppression.^20^ Resection alone led to a decrease in peripheral T cells, accompanied by an increase in CD8^+^ T cells in the bone marrow, which exhibited reduced C-C chemokine receptor 7 (CCR7) expression.^20^ Additionally, granulocytic myeloid-derived suppressor cells (gMDSC) showed a modest increase following resection.^20^

#### Fluorescence-guided techniques

Fluorescence-guided surgery is an advanced intraoperative technique that uses fluorescent agents to enhance the visualization of tumors. These agents preferentially accumulate in cancerous tissues and emit fluorescence when exposed to specific wavelengths of light. Specialized surgical microscopes equipped with dedicated filters are required to visualize this fluorescence in real time, allowing for a clearer distinction between tumor and normal brain tissue. While the overall resection approach remains similar to non-guided freehand techniques, fluorescence reduces the subjectivity of determining where to stop resecting by providing a more objective, real-time guidance. This improves the accuracy of the procedure and minimizes the risk of leaving residual tumor in the brain. However, the technique requires substantial setup time, typically 30 to 60 min, to prepare the microscope and administer the fluorescent agent. Additionally, the need for advanced imaging equipment makes this method significantly more expensive compared to non-guided approaches. In the reviewed articles, two types of fluorescence-guided resections have been identified: cell line-based fluorescence, in which tumor cells are engineered to express fluorescent proteins, and dye-based fluorescence, where exogenous fluorescent agents are administered prior to surgery to label the tumor tissue.^38^^,^^39^

##### Cell-Line-Based Fluorescence-Guided Resection

Most of the authors using fluorescence-guided methodologies relied on the transfection of tumor cells and eleven studies using this technique were included in this review.^10^^,^^22–31^ The transfected cells are then inoculated into the mouse brains (allografts or xenografts depending on the cell lines used) and give rise to spontaneously fluorescent tumors, when illuminated with the proper wavelength and observed via the appropriate filters (Figure 2). The red fluorescent protein (RFP) mCherry is one of the most widely used for transfection. It belongs to a group of chromophore proteins designed for visualization; it absorbs light between 540-590 nm and emits light in the range of 550-650 nm. Another commonly used fluorescent chromophore is the green fluorescent protein (GFP), which emits green fluorescence when exposed to blue to ultraviolet light. GFP absorbs blue light at 395 nm and emits green light at 508 nm.

Two studies by Sheets et al reported intraoperative bleeding during resection, which was managed by irrigation with PBS combined with either “Surgicel” application or steady pressure using a cotton-tipped applicator.^22^^,^^30^ In severe cases, the cavity was temporarily packed with a hemostatic agent.^30^ Two studies reported the use of post-operative care. In Graham-Gurysh et al, mice received subcutaneous carprofen twice daily for 3 days after surgery for pain management.^23^ With Sheets et al, analgesics were administered according to a schedule approved by the Institutional Animal Care and Use Committee (IACUC).^30^ Sheets et al and Bagό et al did not observe any surgery-induced morbidity or mortality.^22^^,^^28^ However, in the study of Fan et al, four out of six resected mice died in the early postoperative period.^26^ To further refine the resection technique, contrast agents were used to visualize blood vessels in two studies: Katta et al injected GFP-FITC-dextran via the tail vein, whereas Kauer et al administered AngioSense-750.^25^^,^^27^ Clear vascular fluorescence was observed; however, this was because of disruption of the blood-brain barrier (BBB).^25^ In most studies, subtotal resection was achieved. Five studies reported the extent of tumor removal: Okolie et al and Hingtgen et al achieved over 90% tumor removal, while Graham-Gurysh et al observed an average resection rate of 95.4%.^23^^,^^24^^,^^31^ Fan et al even reported a resection rate exceeding 99% using a laser-based system to remove the tumor.^26^ In Kauer et al, more than 60% of tumor tissue was resected in mice with small tumors, compared to over 80% in mice with larger tumors.^27^

Five studies reported a significantly prolonged survival of resected mice.^10^^,^^26–28^^,^^31^ Moreover, in the study by Sheets et al, median survival of resected mice was 31 days post-resection.^22^ In addition, Okolie et al showed that following resection, reactive astrocytes co-expressing glial fibrillary acidic protein (GFAP) and nestin increased 16-fold in the peritumoral microenvironment by day 3 and gradually declined thereafter, suggesting that resection alters peritumoral astrocytes and may induce a stem cell-like phenotype.^24^

Seven studies reported tumor recurrence following resection. Sheets et al observed recurrence 3 weeks post-resection by BLI.^22^ In the study by Graham-Gurysh et al, tumors regrew rapidly, as observed by BLI, with all resected mice relapsing within 10 days.^23^ Okolie et al detected recurrent tumors near the resection cavity as early as 5 days post-resection, monitored by serial BLI.^24^ These recurrent tumors grew significantly faster than their pre-resection counterparts and, from 7 days onward, became large, highly mitotically active, and necrotic.^24^ In addition, recurrent tumor cells exhibited extensive perivascular migration and diffuse invasion of the surrounding brain parenchyma.^24^ Hingtgen et al reported that small residual tumor deposits increased markedly in volume from day 7, resulting in multifocal recurrence that progressed into a single large tumor by day 15 post-resection on both BLI and MRI.^31^ Notably, recurrent tumors differed from their pre-resection counterparts, exhibiting larger volumes, due to accelerated growth rates and reduced blood vessel density.^31^ Finally, the studies by Choi et al, Fan et al, and Kauer et al reported recurrence in all resected animals, as monitored by BLI, although no further details regarding relapse were provided.^10^^,^^26^^,^^27^

One study also investigated the effects of resection on the tumor immune microenvironment. Choi et al reported that surgical resection altered the immunosuppressive tumor microenvironment in the NS/CT-2A model by reducing MDSC, enhancing dendritic cell and T-cell infiltration within the resection area, and increasing the M1-like to M2-like macrophage ratio.^10^

##### Dye-Based Fluorescence-Guided Resection

Sodium fluorescein (FL) was used for fluorescence-guided resections in only two preclinical studies, including ours.^32^^,^^33^ This technique has also been successfully applied in patients undergoing brain tumor surgery.^40^^,^^41^ FL is a green fluorescent compound traditionally used in retinal angiography for diagnosing vascular disorders. FL fluorescence is intense, with a peak excitation at 495 nm and peak emission at 520 nm. The initial application of FL for glioma was reported by Dr. George Moore in 1947, demonstrating its potential to provide real-time information about tumor borders.^41^ In more recent times, different authors confirmed feasibility and advantages of FL-guided high-grade glioma resection in patients.^40^ FL is administered intravenously and it usually remains intravascular since the wall of brain blood vessels is not permeable to this molecule. However, due to the leakage of the BBB in the context of malignant brain tumors, FL can extravasate and accumulate in tumor tissue, allowing for the visualization of the tumor mass. In both studies, clear FL fluorescence was seen in the brain tumors.^32^^,^^33^

FL fluorescence can be detected using a surgical microscope equipped with appropriate excitation and emission filters. Typically, a blue light source (around 490-500 nm) is required to excite the FL, and a yellow or green filter is used to enhance the emitted fluorescence around 520 nm. Some modern neurosurgical microscopes, such as the Zeiss YELLOW 560 or the Leica FL560, are designed to optimize FL visualization, allowing surgeons to distinguish tumor tissue from healthy brain structures effectively. Unlike 5-ALA, which produces a red fluorescence visible only under specific violet-blue light, FL emits a bright yellow-green fluorescence that can sometimes be perceived by the naked eye under standard white light conditions, although the use of dedicated light sources and filters significantly improves contrast and visibility (Figure 2).

Ziani-Zeryouh et al reported the use of postoperative care, which was initiated on the day of resection and continued for 2 days thereafter.^32^ This care included intraperitoneal administration of penicillin and buprenorphine twice daily, and a 5% glucose solution once daily.^32^ In addition, soft, high-caloric food was provided ad libitum, and drinking water was supplemented with carprofen and enrofloxacin from the day of resection until 2 weeks postoperatively.^32^ Despite these measures, Ziani-Zeryouh et al reported a resection-related mortality rate of 20%.^32^ In contrast, Le Reste et al did not report anything regarding resection-associated mortality.^33^ In their study, surgical resection alone did not significantly improve survival outcomes.^33^ The resections were subtotal, and all operated mice ultimately succumbed to tumor recurrence.^33^ No significant differences in blood vessel density were found between non-resected and resected conditions.^33^ However, recurrent tumor cells exhibited an increased capacity to infiltrate non-tumorous brain tissue.^33^

Near-infrared (NIR) imaging utilizes specific dyes or contrast agents that emit light when exposed to NIR wavelengths, offering deeper tissue penetration compared to visible fluorescence. This makes NIR imaging particularly useful for visualizing tumors, blood vessels, and lymph nodes during surgery. This technique was used in three studies included in this review.^34–36^

In the study by Li et al, the NIR-probe MMP-750, which targets matrix metalloproteinases (MMP) in GBM, was used for both fluorescence molecular imaging (FMI) and fluorescence molecular tomography (FMT) in GBM-bearing mice.^35^ FMI enabled real-time visualization of tumor margins, while FMT provided three-dimensional spatial information about the tumor.^35^ The probe effectively delineated tumor boundaries, facilitating complete tumor resection.^35^ In the study of Kurbegovic et al and Li et al, the contrast agent IRDye800CW, a near-infrared (NIR) dye that absorbs light at around 778 nm and emits fluorescence at approximately 800 nm, enabled high-resolution imaging and provided real-time guidance for precise tumor detection and surgical resection.^34^^,^^36^ Moreover, IRDye800CW outperformed 5-ALA, as less residual tumor was observed following probe-guided resection.^34^ In addition, BBB penetration was evaluated using a 3D BBB spheroid model, showing probe uptake into the spheroids, indicating its ability to cross the BBB.^34^ Notably, Li et al also evaluated the 68Ga-IRDye800CW-BBN probe, which enables both positron emission tomography (PET) and near-infrared (NIR) imaging, in a cohort of 14 GBM patients, including eight with newly diagnosed and six with recurrent GBM.^36^ The probe demonstrated tumor-specific uptake on PET imaging and provided high-contrast intraoperative fluorescence, allowing accurate tumor delineation and facilitating high rates of near-complete or complete resection.^36^ Among patients with newly diagnosed GBM, a 6-month progression-free survival (PFS) rate of 80% was achieved, substantially higher than the 46% reported with 5-ALA.^36^

None of the studies reported the use of post-operative care. Moreover, no information about post-operative outcomes or recurrence following resection was provided in any of the studies.

#### Magnetic resonance imaging-guided resection

Preoperative MRI combined with neuronavigation is routinely employed in glioma surgery in patients to enhance precision in tumor localization and resection. However, intraoperative MRI is less commonly used in the clinical setting due to its high cost and the additional time required for image acquisition. As a result, other real-time intraoperative techniques, such as ultrasound, are often preferred for guiding resections dynamically.^42^

In this review, we identified only one study reporting the use of MRI to guide tumor resection in a preclinical glioma model.^37^ This approach involved acquiring preoperative T2-weighted MRI scans to delineate the tumor margins and determine stereotactic coordinates for surgical planning (Figure 2). These coordinates were subsequently used during the resection procedure to localize and excise the tumor. Postoperative MRI was then employed to evaluate the extent of resection and to identify potential residual tumor tissue.^37^ Strictly speaking, MRI was not used intraoperatively as it happens for patients. While the pre-postoperative MRI introduces a degree of anatomical precision, it does not incorporate real-time intraoperative guidance. This is a key limitation compared to intraoperative imaging modalities commonly used in clinical neurosurgery and to the other tumor visualization techniques analyzed in this review. The absence of continuous imaging feedback during surgery reduces the capacity to dynamically adapt the resection based on tumor visibility or intraoperative events. Additionally, this technique requires access to pre- and postoperative MRI imaging infrastructure and dedicated surgical planning tools, significantly increasing procedural complexity and cost. The overall duration of the intervention is also prolonged, estimated at approximately 90 min, largely due to the time required for MRI acquisition and coordinate transfer. This makes it the most time-intensive approach among those reviews.

Postoperative care included subcutaneous administration of buprenorphine, with a second dose administered 6 h after resection.^37^ Following surgery, mice exhibited an average body weight loss of 5-10%.^37^ In addition, all animals displayed transient neurological symptoms, including disorientation and circling behavior.^37^ These symptoms resolved completely within 48 h after surgery.^37^ However, two out of 16 mice suffered from respiratory depression probably due to the anesthesia shortly after the resection, and died.^37^

Near-total (80-90%) to complete (∼100%) resection was achieved, as no residual tumor cells were detected by MRI.^37^ Resection significantly prolonged the overall survival of the mice.^37^ Nevertheless, small but detectable tumors were observed on MRI in five mice beginning on day 5 post-resection, and tumor recurrence within the resection cavity ultimately occurred in all animals.^37^ Notably, recurrent tumors exhibited histopathological features and tumor microenvironments similar to those of the primary tumors, with no obvious structural differences.^37^ Both primary and recurrent tumors were associated with ameboid-shaped Iba1^+^ tumor-associated microglia and macrophages, although their density was reduced in recurrent tumors.^37^ In addition, both tumor types showed comparable vascular density, while the average vessel size was significantly larger in recurrent tumors.^37^

## Discussion

In this review, we identified several surgical resection methods in preclinical GBM models. The main features of these techniques are summarized in Table 2.

**Table 2.** Features of GBM resection techniques in mice

Technique	Translational relevance	Advantages	Limitations
Biopsy punch	Low	Fast, easy to perform, consistent cavity size, minimal operator training required	Limited to small, central tumors; fixed cavity size; not representative of clinical surgery
Freehand resection	Moderate	Flexible, allows extent-of-resection assessment, widely used	Highly subjective, requires magnification, variable outcomes, risk of brain damage
Fluorescence-guided resection	High	Enhanced tumor visualization, more accurate resection margins	Expensive equipment, requires expertise and either dye or fluorescent cell lines, potential bias
MRI-guided resection	Moderate	Enables anatomical planning and post-op assessment	No real-time guidance, high cost, complex setup, rarely used

*Note*. Main features of GBM resection techniques in preclinical mouse models.

The biopsy punch method is widely used due to its relative ease and reproducibility, but its fixed resection volume limits its ability to model gross total/partial resection accurately.^12–16^ It also fails to capture peritumoral infiltration and omits the creation of a true surgical margin at the interface between the bulk of the tumor and the infiltrated brain, thereby overlooking postoperative microenvironmental and immune responses that drive recurrence.^43^

Conversely, free-hand resection allows for better customization of the resection according to the research needs, as partial, subtotal and total resections can be mimicked. However, this technique requires much higher surgical skills and more specialized equipment, and its reliance on visual contrast under magnification introduces variability in residual tumor burden and a risk of inadvertent trauma to healthy brain tissue. In non-guided free-hand resection, the amount of tissue removed depends on the researcher’s subjective judgment. As in patients, it is also difficult in mice to clearly distinguish tumor margins from healthy brain, even under microscopic vision. This creates a non-negligible risk of unwanted partial resections. Assessing the impact of these events is further complicated by the lack of standardized reporting of postoperative morbidity and mortality.^17–21^

To overcome this limitation, in line with clinical practice, intraoperative techniques have been developed for preclinical GBM resection in order to enhance tumor visualization. These techniques are mainly based on tumor fluorescence, obtained either by using fluorescent tumor cell lines to generate GBM or by means of preoperative SF injection. Cell line fluorescence is commonly used in other domains in preclinical research, therefore its application in preclinical GBM resection is rather straightforward.^10^^,^  ^22–31^ However, transfecting tumor cells with fluorescent proteins might change their biological behavior and induce antigen production with possible increases in anti-tumor immune reaction (with obvious implications for immunotherapy studies).^44^ Moreover, several studies reported bleeding during resection, which can diminish the intensity of the fluorescent signal and obscure the tumor.^30^ Conversely, preclinical fluorescence-guided GBM resection by means of SF is more adherent to clinical reality. While 5-ALA-based resection remains the most widely used fluorescence-guided technique in GBM surgery, clinical studies have shown that SF-guided resection is an effective alternative.^6^^,^^36–39^ Compared to 5-ALA, SF is less expensive and it requires less sophisticated microscopes to be visualized, making it a good option for preclinical GBM resection. However, SF also shows disadvantages such as the need for intravenous administration, lower tumor specificity compared to 5-ALA, and variable dye uptake that can complicate margin assessment.

In clinical practice, intraoperative imaging such as intraoperative magnetic resonance imaging (iMRI), intraoperative computed tomography (iCT), and intraoperative ultrasonography (iUS) can be used during GBM removal. Intraoperative imaging refers to techniques that provide real-time images while the surgery is ongoing, allowing the surgeon to adjust the extent of resection based on tumor boundaries and anatomy. Our review identified only one study describing the use of imaging to improve tumor resection, in particular MRI. However, in this study, MRI was used pre-operatively to identify tumor location and postoperatively to evaluate the extent of resection. Therefore, this MRI application cannot be assimilated to an actual iMRI where the imaging technique is used during surgery to guide the procedure, and it fails to model the dynamic, real-time decision-making and tissue response monitoring that are hallmarks of clinical image-guided neurosurgery, while also imposing significant logistical and cost barriers.^37^

It is important to note that in the studies included in this review, post-operative morbidity, mortality, and the use of specific operative and postoperative care protocols (e.g. anesthesia and analgesia and humane endpoints) are frequently underreported. Considering that high surgical mortality is a well-documented issue in murine models of GBM resection, systematic reporting of these parameters would be valuable to enable objective comparisons between different surgical techniques and to identify best practices for postoperative management.^45^ Such data could facilitate the development of standardized, optimized pre- and postoperative care protocols aimed at improving both animal welfare and experimental reliability. In this context, our group recently developed and published an intensive postoperative care protocol, which demonstrably reduces surgical mortality and improves animal welfare, thereby enhancing the ethical and cost-effectiveness profile of preclinical GBM resection studies.^46^

Several considerations have to be taken into account when evaluating surgical techniques in preclinical models. First, the choice of cell line is crucial.^47^ Several studies included in this review used the U87 cell line, which fails to reflect the features of human GBM. Moreover, because U87 tumors form well-defined, non-invasive masses, this model makes it relatively easy to excise nearly the entire tumor. In addition to the choice of cell line, the selection of an appropriate animal model should be carefully tailored to the specific research question.^47^ Both immunocompetent and immunocompromised models, which allow implantation of patient-derived GBM cells, were used in the preclinical studies included in this review. Although immunocompromised models enable the study of patient-derived GBM cells, they cannot be used to investigate tumor-immune system interactions, meaning that no preclinical model currently allows patient-derived GBM cells to be studied in mice with an intact immune system. Recently, humanized mouse models, in which components of the human immune system are established in mice, have become available. These models permit investigation of immune responses in patient-derived tumors, including GBM. However, they remain costly and relatively understudied.^47^ Moreover, the location of tumor implantation may influence surgical resection techniques and outcomes.^47^ This factor may be highly relevant, given the substantial variability in tumor location observed in patients. However, it has not yet been investigated in preclinical studies.

Despite its essential role in the treatment of GBM patients, with the extent of resection strongly correlating with outcome, surgical resection is rarely incorporated into preclinical GBM studies.^48–53^ Extent of resection, which is well established as a prognostic factor in glioma, was likewise not reported in any of the included studies. This omission is particularly striking considering the clinical translation and dynamic nature of glioblastoma. Tumors evolve in response to therapy, and recurrent GBMs (after the whole SoC, including surgical resection) are known to diverge significantly in their molecular profile, microenvironment, and immune landscape compared to treatment-naïve tumors.^54^ Surgical intervention initiates a cascade of biological responses that significantly alter the tumor and its microenvironment. The act of resection introduces mechanical disruption to brain tissue, resulting in local inflammation, blood-brain barrier breakdown, and the release of damage-associated molecular patterns (DAMPs). These factors collectively modulate the immune landscape of the brain, recruiting innate immune cells such as microglia and peripheral macrophages, and potentially altering the activation state of resident immune populations.^55^ Moreover, the resection cavity itself becomes a unique ecological niche. Tumor cells that survive the procedure are subject to hypoxia, altered nutrient gradients, and changes in interstitial fluid dynamics. These surviving cells may adopt more aggressive, invasive, or therapy-resistant phenotypes, contributing to recurrence. The exposed edges of the resection margin, where infiltrative tumor cells remain, also represent a key therapeutic target that is absent in non-resected models. Consequently, the response of these residual cells to subsequent therapies may differ substantially from the response of untreated bulk tumors in conventional preclinical designs.^56^ In addition, surgery can affect tumor vasculature and alter drug delivery dynamics. Post-surgical changes in perfusion and permeability influence the pharmacokinetics and bioavailability of administered therapies. The immune contexture also shifts after surgery, with local cytokine production and immune cell trafficking patterns potentially reshaping the tumor-immune interplay. These aspects are crucial when evaluating immunotherapies or treatments that rely on an intact immune system.^57^^,^^58^ The impact of tumor resection on the immune system remains poorly studied, and only a limited number of the included studies address this aspect.^47^ Notably, considerable discrepancies exist among their findings. While Chen et al reported that tumor resection resulted in a more immunosuppressive tumor microenvironment (TME) due to an influx of immunosuppressive macrophages, Choi et al found that resection led to a less immunosuppressive TME.^10^^,^^17^ Similarly, Otvos et al observed a reduction in gMDSC following resection.^20^ Consequently, there is a clear need to investigate how surgical resection shapes the tumor immune microenvironment in preclinical models. By failing to account for all these surgery-induced changes, preclinical models may overestimate the efficacy of experimental therapies. As a result, treatments that appear highly effective in static, non-resected models often fail in clinical trials, where they encounter a more complex and evolving post-surgical tumor environment. Thus, the absence of tumor resection in most translational studies likely represents a critical missing piece in the effort to bridge the gap between preclinical promise and clinical success.^59^^,^^60^

The limited integration of surgical resection in preclinical glioblastoma research can be attributed to several practical, technical, and translational challenges. Performing surgery in (small) animal models requires specialized microsurgical expertise, dedicated equipment, and extensive animal handling. This increases procedural complexity, costs, and ethical concerns. Moreover, differences in surgical training and operative protocols can lead to variability in the extent of resection, potentially compromising the reproducibility of results.^47^ Therefore, accurate assessment of resection margins and completeness using postoperative imaging modalities, such as luminescence imaging, PET/CT, and MRI, is essential to standardize surgical interventions and reduce outcome variability.^47^ Standardizing post-operative care protocols across laboratories adds another layer of difficulty. Additionally, the variability in tumor location, size, and infiltration patterns in preclinical models makes it challenging to develop reproducible and clinically relevant resection techniques. As a result, at present the majority of GBM preclinical studies rely on a simpler, less demanding approaches that exclude surgery, despite its pivotal role in the clinical management of GBM.^61^

Our review shows that incorporating tumor resection surgery in preclinical GBM studies is feasible via different strategies. However, as already stated, in most cases GBM patients undergo a more complex SoC including also radiotherapy and chemotherapy.^62^ Our group recently demonstrated that the implementation of a complete SoC is possible in GBM mouse models and that, as for patients, also for GBM-baring mice survival is longer when the complete standard of care is administered compared to adjuvant treatments alone.^46^ Other studies have shown that combining resection with local drug delivery systems, such as hydrogels, significantly prolongs survival in murine models by ensuring sustained therapeutic exposure in the post-operative cavity.^13–17^^,^^19^^,^^21^^,^^22^^,^^23^^,^^27^^,^^28^ Therefore, implementing in preclinical studies the full clinical SoC represents the obvious next step to increase the predictive value of preclinical studies.

## Conclusion

Surgical resection is a cornerstone in the clinical management of GBM and has a significant impact on patient outcomes. However, it remains underutilized in preclinical GBM research. This review highlights that surgical tumor removal in mice is both feasible and effective, with several techniques available in the literature. We advocate for a more systematic incorporation of surgical resection into preclinical GBM studies to enhance their translational relevance and better reflect the clinical setting.

## Data Availability

All data supporting this review are included in the article.
